# SKELETAL MUSCLE HEALTH AND LOWER URINARY TRACT SYMPTOMS IN OLDER ADULTS

**DOI:** 10.1093/geroni/igac059.2599

**Published:** 2022-12-20

**Authors:** Peggy Cawthon, Scott Bauer, Candace Parker-Autry, Kaiwei Lu, Steven Cummings, Russell T Hepple, Rebecca Scherzer, Kenneth Covinsky

**Affiliations:** California Pacific Medical Center Research Institute, San Francisco, California, United States; UCSF and San Francisco VA, San Francisco, California, United States; Wake Forest School of Medicine, Winston-Salem, North Carolina, United States; San Francisco VA, San Francisco, California, United States; San Francisco Coordinating Center, San Francisco, California, United States; University of Florida, Gainesville, Florida, United States; San Francisco VA, San Francisco, California, United States; University of California, San Francisco, San Francisco, California, United States

## Abstract

Lower urinary tract symptoms (LUTS) are associated with increased risk of mobility limitations among older adults. Our objective was to evaluate the association of muscle (D3Cr muscle mass, MRI total thigh muscle volume, Keiser extensor power, grip strength) and physical performance (400m walk, SPPB) measures with LUTS severity and bother among adults age >70 years in the Study of Muscle, Mobility and Aging (SOMMA). We used data from the first 132 women and 103 men to complete their baseline visit where LUTS were assessed using the LURN Symptom Index-10 (SI-10) plus a global urinary bother question. We calculated Spearman correlation coefficients and chi-square tests as appropriate, stratified by sex. Among women, LURN SI-10 scores were inversely correlated with D3Cr muscle mass/body weight (ρ=-0.217, P=0.01), peak leg power/body weight (ρ=-0.179,P=0.04), and SPPB (ρ=-0.173,P=0.047), but not 400m walk, MRI thigh muscle volume, or grip strength (P>0.1 for all). 46% of women in the lowest tertile of % muscle mass versus 38% in the highest tertile reported they were at least “somewhat bothered” by urinary symptoms (P=0.04). Among men, no muscle or physical performance measures were significantly associated with LURN SI-10 or urinary bother (P>0.2 for all). In conclusion, older women with greater muscle mass, leg power, and SPPB scores had reduced LUTS severity whereas LURN SI-10 was not significantly correlated with muscle and physical performance measures in older men. Older women with higher D3Cr muscle mass were also less bothered by urinary symptoms, supporting muscle health as a novel female LUTS mechanism.

